# Improving the Adjuvanticity of Small Molecule Immune Potentiators Using Covalently Linked NF-κB Modulators

**DOI:** 10.1021/acsmedchemlett.1c00267

**Published:** 2021-08-26

**Authors:** Flora W. Kimani, Saikat Manna, Brittany Moser, Jingjing Shen, Naorem Nihesh, Aaron P. Esser-Kahn

**Affiliations:** Pritzker School of Molecular Engineering, University of Chicago, Chicago, Illinois 60637, United States

**Keywords:** Small molecule immune potentiators, adjuvants, NF-κB

## Abstract

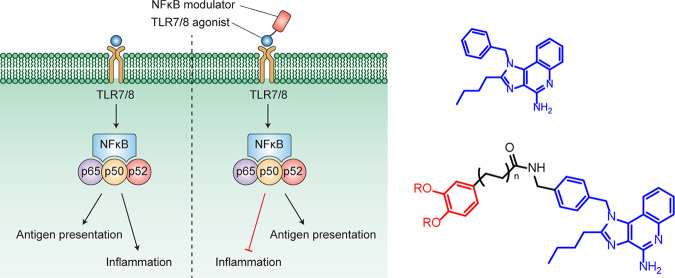

Small molecule immune potentiators (SMIPs) such as imidazoquinolinone derivatives that activate Toll-like receptor (TLR) 7/8 have immense potential as vaccine adjuvants and as antitumor agents. However, these molecules have high bioavailability that results in unacceptable levels of systemic inflammation due to adjuvant toxicity, thereby greatly limiting their use. To address this challenge, here we report the design and synthesis of novel imidazoquinolinone-NF-κB immunomodulator dimers. Employing *in vitro* assays, we screened a select library of synthesized dimers and selected viable candidates for further *in vivo* experiments. With ovalbumin as a model antigen, we vaccinated mice and demonstrated that these dimers reduce the systemic toxicity associated with SMIPs to baseline levels while simultaneously maintaining the adjuvanticity in a vaccine formulation. Additionally, we showed that select dimers improved efficacy in a CT26 mouse colon carcinoma tumor model while eliciting minimal adjuvant toxicity.

Toll-like receptor (TLR) activation in innate immune cells has been linked to the high immunogenicity and protective effects of vaccines.^1,2^ The incorporation of TLR activating immunostimulants or adjuvants in subunit and epitope-based vaccine formulations has led to great improvements in both antibody and T-cell levels and antigen specificity.^3,4^ Currently, the discovery of many small molecule adjuvants has demonstrated immense potential and opened up the possibility for new therapies.^5,6^ However, their tolerability in preclinical and clinical studies has limited the use of many of these compounds requiring either reformulation or redesign.^7^ Historically, discovery of adjuvants has been empirical, but with synthetic small molecule adjuvants, modern drug discovery techniques permitted optimized adjuvanticity. This opportunity has led to the development of a class of adjuvants collectively referred to as small molecule immune potentiators (SMIPs).^8,9^ In this class, imidazoquinolinones that activate TLR 7/8 such as imiquimod (R837) and resiquimod (R848) have been extensively studied. Imiquimod is currently approved for clinical immunotherapy use in topical creams.^9−11^ These SMIPs elicit antigen specific cellular responses when administered as adjuvants.^12−14^ Additionally, activation of TLR7/8 by resiquimod can lead to antitumor activity facilitated by APC activation of CD8+ T cells and CD4+ Th1 cells due to IFN-γ and IL-2 production and hence enhanced proliferation.^15−17^ Unfortunately, despite such tremendous potential, the high bioavailability of imidazoquinolinone adjuvants and associated toxicity has primarily limited the use of TLR7/8 agonist formulations to topical applications and prevented their approval as injectable adjuvants in humans.^9,18,19^ To address this challenge, here we report on an alternative method that links a TLR7/8 activating imidazoquinolinone to an NF-κB modulator, thereby modulating the response to the molecule directly elicited from immune cells (Figure 1).

**Figure 1:**
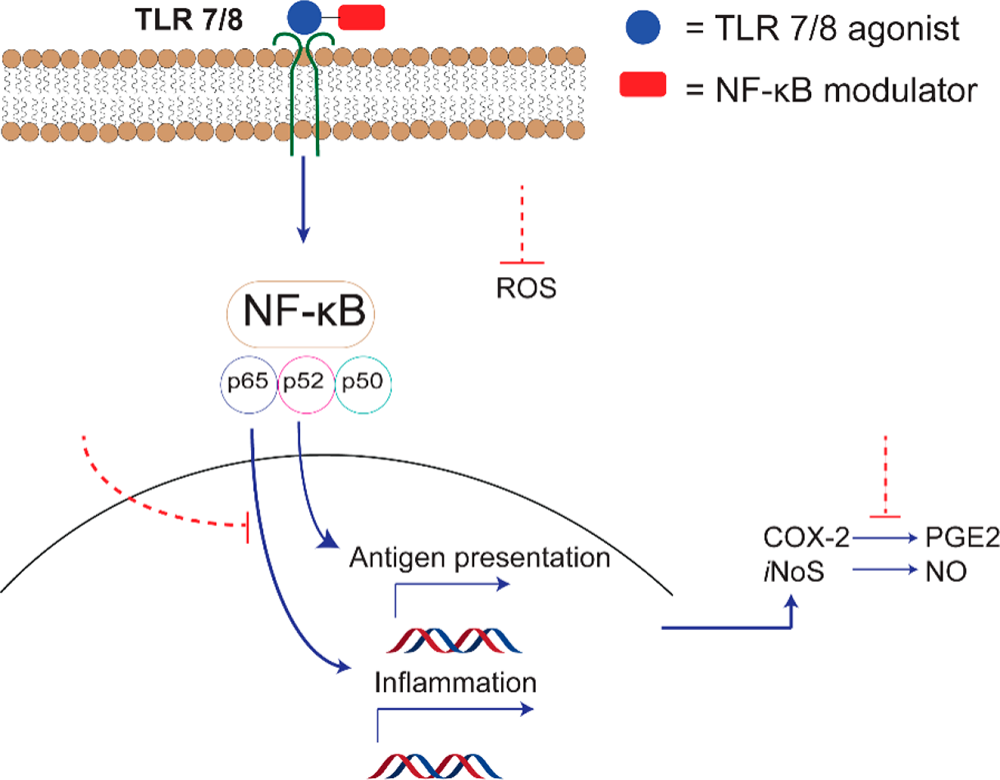
Comparing methods of small molecular immune potentiation. Traditional SMIP adjuvant NF-κB pathway. Activation of TLR7/8 with the agonist (blue) leads to downstream pro-inflammatory responses and responses associated with adaptive immunity (antigen presentation). In our work, the adjuvant contains an activation moiety and a direct modulation moiety which alters the NF-κB pathway. Activation of TLR7/8 with the dimers also results in activation of the NF-κB pathway by the adjuvant (blue), while the tethered modulators (red) inhibit specific parts of the pathway resulting in lower pro-inflammatory immune response and enhanced or unchanged adaptive immune response.

Recently, we reported that small molecule NF-κB inhibitors can be used to modulate the activity of CpG (TLR 9 agonist) in vaccine formulations. In a screen, we showed that capsaicin and honokiol reduced pro-inflammatory systemic IL-6 and TNF-α levels while maintaining vaccination efficacy.^20^ Unfortunately, we did not observe similar effects with *in vivo* experiments employing TLR7/8 agonist R848 as an adjuvant. We hypothesized that this was due to the high rate of diffusion of the small molecule adjuvant and the immune modulators from the injection site. Therefore, we designed hybrid molecules by covalently linking a imidazoquinolinone derivative^21^ with a conjugatable amine handle to vanilloid, catechol, and honokiol^20^ derivatives. We envisioned such a construct would lead to simultaneous cellular coactivation of TLR 7/8 receptors simultaneous with immunomodulation from the coupled NF-κB modulators. We ran *in vitro* experiments using murine macrophages and identified candidates to test in *in vivo* experiments. Using ovalbumin (OVA) as a model antigen, we demonstrate that the imidazoquinolinone-immune modulator dimers significantly reduce systemic toxicity induced by the small molecule adjuvant while maintaining the adjuvanticity in vaccine formulation. We also investigated imidazoquinolinone antitumor activity by employing a CT26 mouse colon carcinoma tumor model and introduced the dimers through peritumoral injection. We showed that select dimers increased mouse survivability by inhibiting tumor proliferation while inducing low systemic inflammation and reducing adjuvant toxicity. With these results, we show that by tethering NF-κB modulators to SMIPs we improve their tolerability without affecting adjuvanticity and antitumor efficacy.

## Results and Discussion

### Synthesis of Imidazoquinolinone-Immune Modulator Dimers

**Figure 2:**
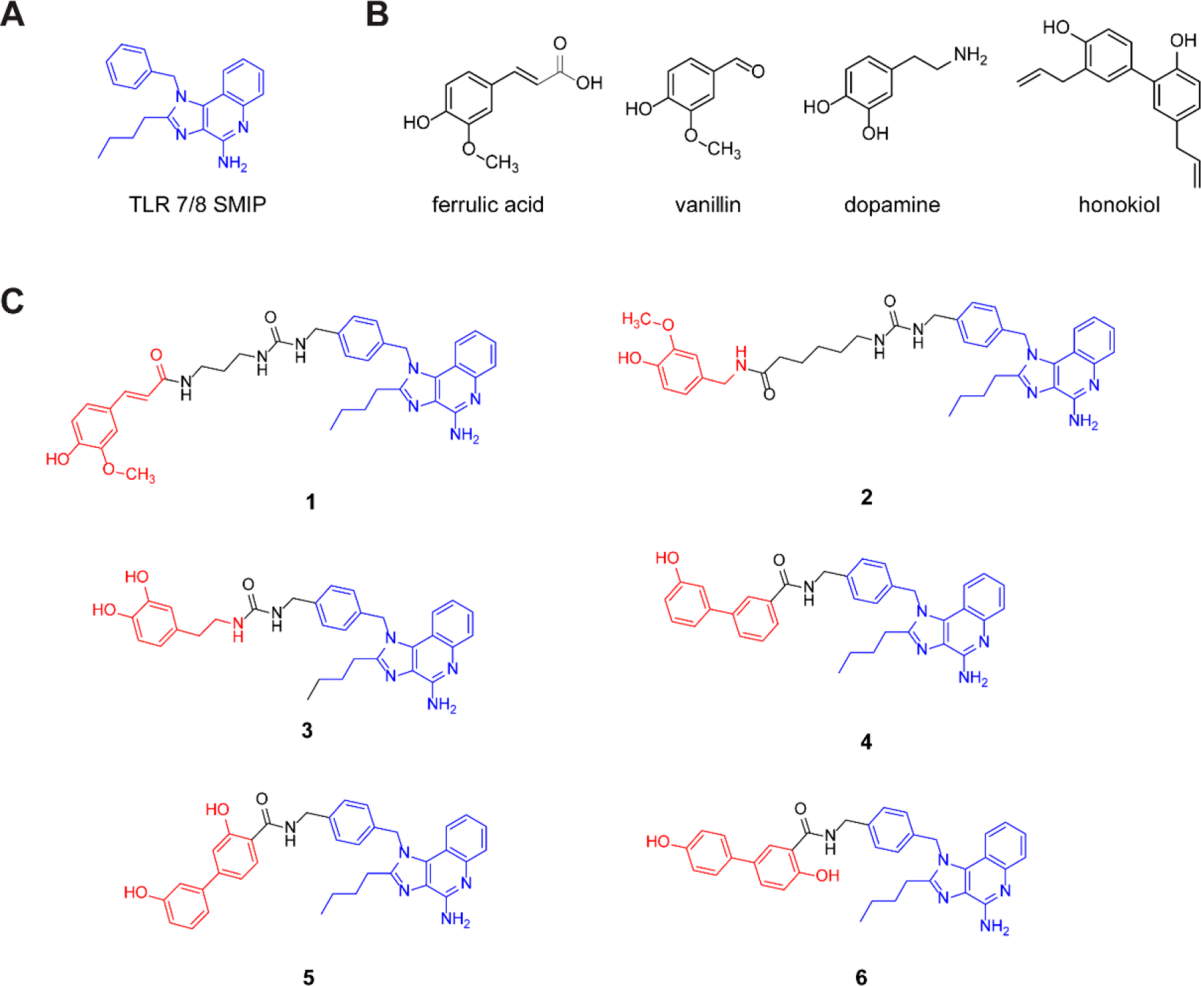
A) TLR 7/8 small molecule potentiator (SMIP) imidazoquinolinone, B) NF-κB small molecule modulators, and C) synthesized (blue) TLR 7/8_NF-κB modulators (red) dimers.

In our previous studies using small molecule NF-κB modulators in vaccine formulations, we screened various commercially available molecules both *in vivo* and *in vitro*. From this screen, we identified vanillin and honokiol derivatives as the most effective small molecule modulators.^20^ These anti-inflammatory and antioxidant molecules have been extensively studied in the literature for NF-κB modulation through direct inhibition of the canonical NF-κB pathway or through scavenging pro-inflammatory mediators such as nitric oxide and other ROIs.^22−24^ However, when we performed *in vivo* experiments using a mixture of R848 and capsaicin or honokiol modulators in vaccine formulations, we observed high systemic cytokines 1 h after vaccination (Figure S1). For this work, we designed and synthesized dimers by conjugating a TLR 7/8 imidazoquinolinone derivative with a conjugatable amine handle^21^ to vanillin, catechol, and honokiol derivatives to yield IMD-ferulic (**1**), IMD-vanillin (**2**), IMD-catechol (**3**), IMD-biphenylA (**4**), IMD-biphenylB (**5**), and IMD-biphenylC (**6**) (Figure 2).

### *In Vitro* Analyses of Synthesized Dimers

**Figure 3:**
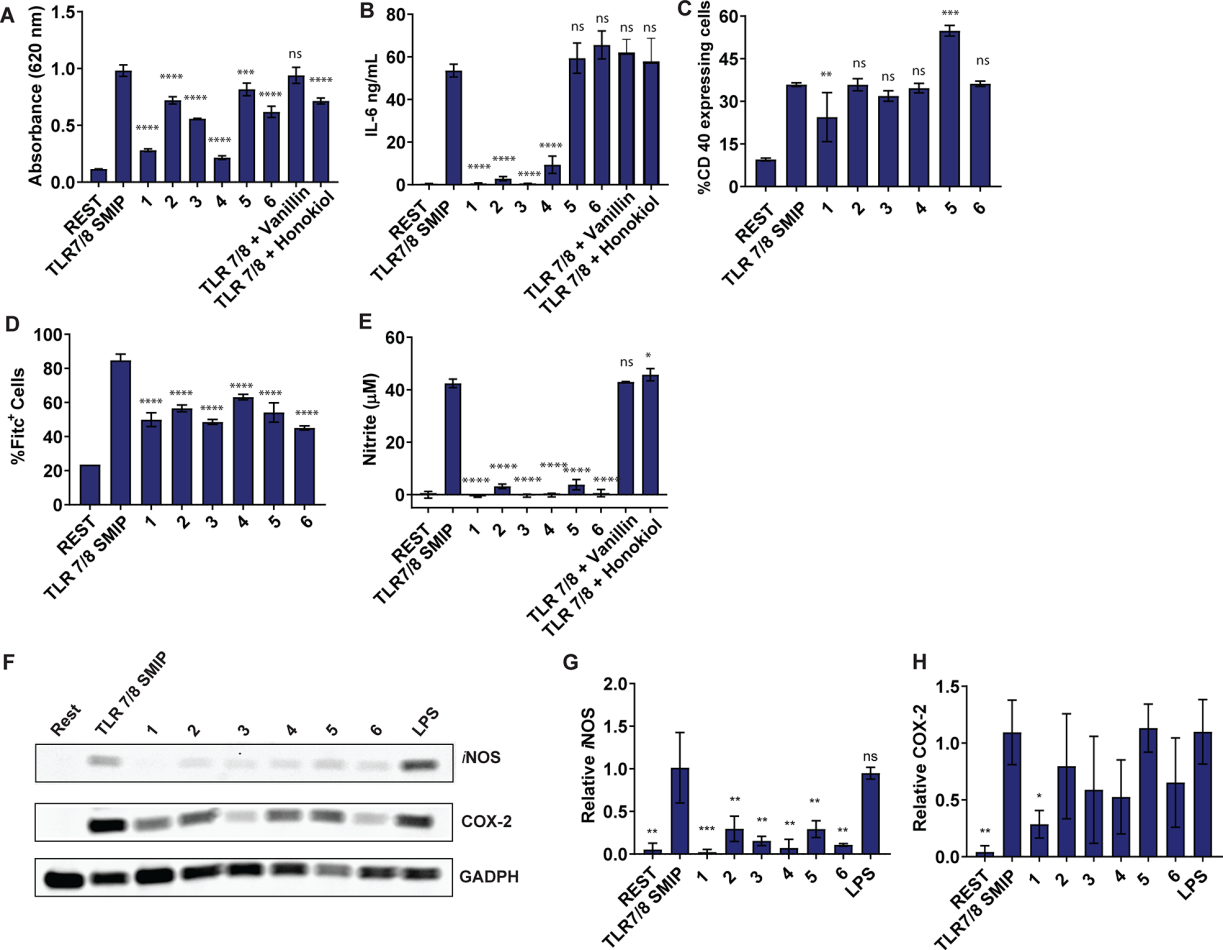
*In vitro* assays determining TLR 7/8 NF-κB activation and modulation of synthesized dimers. A) Immune activation measured by RAW-Blue activation via NF-κB stimulation after 24 h incubation with 500 nM of compounds at 37 °C. B) IL-6 expression (ELISA) and C) cell surface protein expression (FACS) measured 8 h after incubation with BMDCs. Compounds assayed at 200 nm. D) Intracellular reactive oxygen species (ROS) measured by incubating RAW macrophages with 6-chloromethyl-2′,7′-dichlorodihydrofluorescein diacetate, acetyl ester (CM-H_2_DCFDA) and fluorescence measured using flow cytometry. E) Nitrite levels in supernatant of RAW macrophages incubated with 500 nM of compounds for 16 h and measured using Griess reagent. F,G) Expression of COX-2 protein measured in the lysate of RAW macrophages incubated with 500 nM compounds for 16 h. Samples run in triplicate. Statistical significance to TLR 7/8 SMIP, compared by the one-way ANOVA *p ≤ 0.05, **** *p* ≤ 0.0001 A+B denotes equimolar mixture. IMD-ferulic (**1**), IMD-vanillin (**2**), IMD-catechol (**3**), IMD-biphenylA (**4**), IMD-biphenylB (**5**), and IMD-biphenylC (**6**).

Next, we designed an *in vitro* screen to test the synthesized dimers and identify promising candidates for further development in an *in vivo* model. Using a RAW macrophage NF-κB-SEAP (Secreted Alkaline Phosphatase) reporter cell line, we measured the overall activity of the compounds. We saw a reduction in activity of the dimers compared to the imidazoquinolinone agonist and equimolar mixtures of the agonist and NF-κB small molecule modulators (Figure 3A). We next proceeded to analyze if the reduction in activity is due to disruption of cellular uptake and receptor binding or due to selective modulation of NF-κB activity. To elucidate the activity of the dimers, we ran pro-inflammatory cytokine and cell surface protein expression assays on murine bone marrow derived dendritic cells (BMDCs). After incubating the parent SMIP and the dimers with BMDCs for 8 h, we observed that dimer compounds IMD-ferulic (**1**), IMD-vanillin (**2**), IMD-catechol (**3**), and IMD-biphenylA (**4**) reduced the levels of IL-6 secreted to almost baseline levels. Compounds IMD-biphenylB (**5**) and IMD-biphenylC (**6**) did not significantly change the IL-6 levels when compared to the parent SMIP. We also observed that equimolar mixtures of the SMIP and the small molecule NF-κB modulators did not lower the levels of IL-6 secreted (Figure 3B). In a similar BMDC experiment, we stained the BMDCs for cell surface expression of CD40, a well characterized costimulatory molecule with an important role in adaptive immunity^25^ and quantified the expression levels using flow cytometry. Notably, we observed that the expression levels of CD40 remained unchanged for most of the compounds and was slightly lower for IMD-ferulic (**1**) (Figure 3C). Using this experiment we screened for dimers that would lower pro-inflammatory cytokines while maintaining or improving cell surface protein expression. This would indicate that the hybrid molecule was modulating the NF-κB response of the imidazoquinolinone as opposed to merely inhibiting the activity.

In addition to inhibition of the upstream events of the NF-κB pathway, the small molecule modulators we selected to derivatize and synthesize the adjuvant dimers were previously described in the literature as downstream inhibitors of pro-inflammatory mediators such as nitric oxide and reactive oxygen species (ROS).^24^ We were interested in learning if the dimer adjuvants would have a similar effect on immune cells. To study the effect of the dimers on oxidative stress, we incubated RAW macrophages with the compounds for 16 h and measured the levels of intracellular ROS using ROS-reactive fluorescent dye, CM-H_2_DCFDA and quantified the fluorescence using flow cytometry. Here, we observed a nearly 50% reduction in intracellular ROS for all the dimer agonists suggesting that these compounds were ROS scavengers (Figure 3D). In a similar experiment, we incubated RAW macrophages for 16 h with the dimer agonists and used Griess reagent to measure the levels of nitrite, a metabolite of nitric oxide in the cell supernatant. The nitrite levels were quantified by measuring absorbance using a plate reader. From this experiment, we showed that the dimer adjuvants reduced the NO levels to baseline levels compared to the parent SMIP (Figure 3E) indicating that the dimers were potent inhibitors of nitric oxide either through scavenging or direct inhibition of the enzymatic pathway. Additionally, using Western blot analysis of RAW macrophage cell lysate we observed that the molecules inhibited inducible nitric oxide synthase (*i*NOS), a pathway precursor of NO (Figure 3F,G).

Lastly, we wanted to investigate if the dimer agonists were cyclooxygenase-2 (COX-2) inhibitors. COX-2 is a pro-inflammatory marker associated with the activation of both the NF-κB and MAPK pathways. After incubating RAW macrophages with the dimer agonists for 16 h, we lysed the cells and separated the proteins using SDS-PAGE after which we transferred the proteins to a membrane and probed the protein levels using an anti-COX-2 antibody. We observed low relative expression levels of COX-2 protein with IMD-ferulic (**1**). However, IMD-vanillin (**2**), IMD-catechol (**3**), IMD-biphenylA (**4**), IMD-biphenylB (**5**), and IMD-biphenylC (**6**) did not significantly reduce COX-2 expression suggesting that these compounds were not inhibitors of the COX-2 pathway (Figure 3F,H).

### *In Vivo* Analysis of Synthesized Dimers

**Figure 4:**
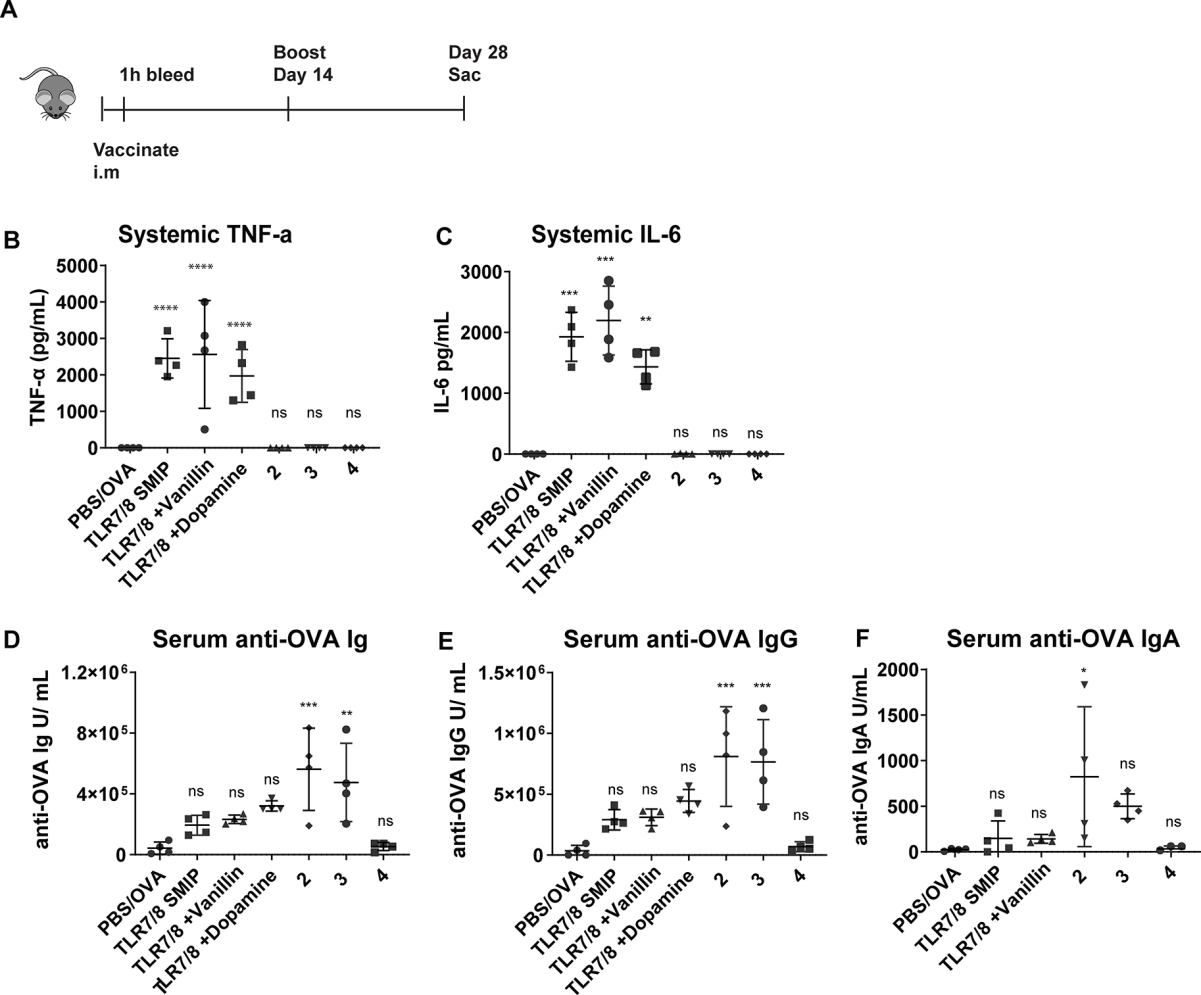
*In vivo* assays TLR 7/8_NF-κB modulator dimers. A) Outline of *in vivo* vaccination experiment. B) Serum levels of cytokines assayed 1 h after injection. IL-6 and TNF- α levels of linked compounds not detected. C) Serum anti-OVA antibodies measured day 28. Statistical significance to a PBS control, compared by the one-way ANOVA *p ≤ 0.05, **** *p* ≤ 0.0001 A+B denotes equimolar mixture. IMD-vanillin (**2**), IMD-catechol (**3**), IMD-biphenylA (**4**).

With this promising *in vitro* analysis, we next set up *in vivo* experiments to see how these dimers would perform in a vaccine formulation. Using ovalbumin as a model antigen, we vaccinated mice with the most promising dimers (IMD-vanillin (**2**), IMD-catechol (**3**), and IMD-biphenylA (**4**)) that reduced inflammation while maintaining CD40 expression in the *in vitro* assays (Figure 3B,C). IMD-ferulic (**1**) was structurally similar to IMD-vanillin (**2**) and was slightly less effective at inducing expression of CD40 compared to the parent agonist (Figure 3C). Compounds IMD-biphenylB (**5**) and IMD-biphenylC (**6**), while activating CD40, were not effective at reducing inflammation (Figure 3b) and were therefore excluded from further *in vivo* experimentation. We performed intramuscular injection (i.m.) with 100 μg of OVA, 70 nmol of imidazoquinolinone, potentiated dimers, and equimolar mixtures of imidazoquinolinone and NF-κB modulators in 50 μL of PBS. At the 1-h mark postinjection, we collected serum from the mice and quantified systemic levels of TNF-α and IL-6. We observed that the dimers reduced these systemic cytokines to background levels comparable to the PBS, vanillin, and catechol controls (Figure 4B,C). On day 14, we performed a boost injection, and then on day 28, we sacrificed the mice, collected sera, and analyzed anti-OVA antibodies. Notably, compounds IMD-vanillin (**2**) and IMD-catechol (**3**) induced significantly higher levels of anti-OVA Ig (A+G+M) and specific IgG compared to the TLR7/8 adjuvanted mice (Figure 4D,E). Comparing specific IgA antibodies, we saw that IMD-vanillin (**2**) induced statistically higher levels of these antibodies (Figure 4F).

Because activation of TLR 7/8 has been previously associated with increased CD8+ T cell function,^17^ we were interested in investigating this activity for these new compounds compared to the parent TLR 7/8 agonist. On day 28 post OVA *in vivo* vaccination experiment, we harvested the spleen of the mice and prepared a single cell splenocyte suspension. We then incubated the cells with an SINFEKL MHC specific tetramer and analyzed the cells using flow cytometry. We did not observe increased activity with the agonist dimers compared to the vehicle control (Figure S3A). This result was expected because this route of administration, admixing OVA and the adjuvant in PBS and i.m. injection, has been reported to enhance antibody titers but not T-cell immunity.^26^ In a separate experiment, we examined splenocyte proliferation with naïve splenocytes, incubating the cells for 48 h with the dimer agonists and parent adjuvant. We found that the dimer agonists promoted the proliferation of lymphocytes to comparable levels when compared to the parent agonist (SI Figure 3B). In summary, the attachment of the modulator slightly increased the activity of the parent compound to generate antibody responses while maintaining all other aspects of its innate immune stimulation except excess cytokine production.

### Antitumor Activity of Dimer Agonists

With the promising results from our preliminary vaccination experiments, we were further motivated to evaluate their efficacy as cancer immunotherapeutics. Imidazoquinoline SMIPs have shown great promise as antitumor immune-therapeutic agents, but their inflammation has often hindered effective therapeutic development. To examine how adding NF-κB modulation to a SMIP might alter this, we tested the antitumorigenic activity of the dimers. Previous studies have shown that intratumoral adjuvant introduction is effective in reducing tumor proliferation by enhancing T-cell antitumor activity.^27^ Specifically, TLR 7/8 activation leads to enhanced innate immune cell activation propagated by increased secretion of IFNα, IL-12, and IFN-γ cytokines.^17,28^ For most compounds used for this purpose, especially resiquimod (R848), systemic adjuvant toxicity is a major limiting factor. As our dimer agonist platform reduced systemic cytokines, we decided to test the compounds on a tumor model. In this *in vivo* model, we used the CT26 tumor models and administered the adjuvant and select adjuvant dimers (compounds IMD-ferulic (**1**), IMD-vanillin (**2**), IMD-catechol (**3**), IMD-biphenylA (**4**), and IMD-biphenylB (**5**)) and a PBS control. Additionally, we included controls, resiquimod (R848) along with a resiquimod derivative 3M-052, that have been employed in clinical trials of cancer immunotherapy.^28^

**Figure 5:**
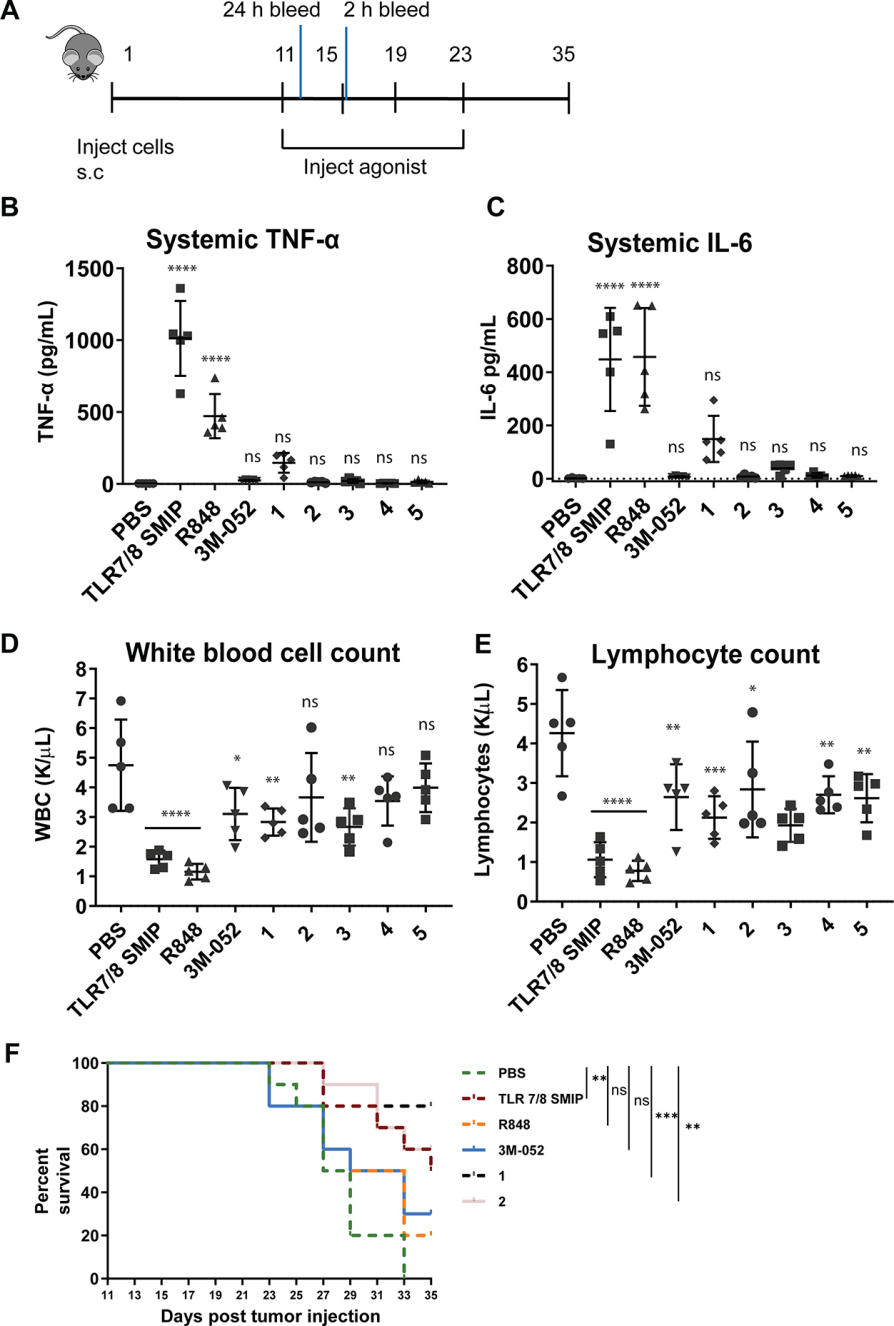
A) *In vivo* tumor model experiment using SMIP-modulator dimers with peritumoral injection into a subcutaneous CT-26 tumor model. Agonists were injected when tumors were about 75 cc in size followed by three additional injections every 4 days. B) Systemic cytokine levels measured in the serum 2 h after a peritumoral injection of compounds. D,E) Hematological analysis of peripheral blood measured 24 h after an intertumoral injection of compounds. F) Kaplan–Meier survival analysis-tumor size was measured every alternate day, and animals were euthanized when the tumors reached 20 mm in any linear dimension. Measurement was performed until day 35. The tumor volume was measured using the formula 0.5*L*W*W. Statistical analysis was conducted using the logrank test with the Bonferroni correction.

After 11 days, the tumors were established, and we administered the adjuvants and adjuvant dimers via peritumoral injection. We collected serum and blood, at 2 and 24 h after these injections, respectively, to measure systemic cytokines in the serum and quantify adjuvant toxicity via a hematological analysis on the blood. Similar to the OVA vaccination model, we observed that the dimer adjuvants induced baseline levels of TNF-α and IL-6 as measured in the serum (Figure 5B,C). In contrast, the parent adjuvant and R848 induced high levels of these pro-inflammatory cytokines, while 3M-052 was comparable to the vehicle control and the dimer adjuvants. Interestingly, IMD-ferulic (**1**) induced slightly elevated IL-6 and TNF-α when compared to the rest of the tested dimer adjuvants. Additionally, the hematological analysis of the blood showed that the molecules that induced higher systemic inflammatory cytokines led to lower white blood count and lymphocyte counts compared to the vehicle control (Figure 5D,E). These results suggest that the parent adjuvant and R848 upon peritumoral injection resulted in much higher adjuvant induced toxicity.

Observing promising tolerability with the adjuvant dimers, we continued the tumor model experiment, monitoring tumor proliferation. Since CT-26 is a highly aggressive murine carcinoma model, tumor proliferation was monitored until day 35. We observed that of the dimer adjuvants tested, two dimer molecules, IMD-ferulic (**1**) and IMD-vanillin (**2**), improved survivability with IMD-ferulic (**1**) providing 80% survivability at day 35 which was higher than the parent adjuvant, 3M-052 and R848 (Figure 5F, see SI Figure 4 for tumor growth curves). We observed slightly elevated levels of IL-6 and TNF-α in the serum of mice injected with IMD-ferulic (**1**) suggesting moderate levels of systemic immune activation by these molecules might be necessary for efficacious responses. Because it has never been possible before to decouple the inflammatory response from the activity of a specific molecule, it is possible that the improvement we observe is the cumulative effect of reduced inflammation while maintaining identical T-cell responses. Overall, this study indicates that our dimer agonists serve as a powerful platform to reduce toxicity of SMIP agonists while maintaining or enhancing efficacy.

## Conclusion

Agonists that activate TLR7/8 receptors are an attractive source of vaccine and cancer immunotherapy adjuvants. These receptors are broadly expressed on antigen-presenting cells and once activated lead to enhancement of APC maturation with increased expression of costimulatory markers and cytokine expression which causes both cellular and humoral immunity. SMIPs activating TLR 7/8 such as the imidazoquinolinone family are potent adjuvants whose major limitation is unfavorable systemic toxicity limiting their use. In this study, we show that by chimeric assembly of these adjuvants with a NF-κB potentiating small molecule we can modulate these molecules, reducing the unfavorable toxicity. We show using an *in vitro* screen how to identify viable dimers and demonstrate their adjuvanticity in a vaccination model as well as antitumorigenic activity without systemic toxicity. Due to the large availability of molecules that modulate the NF-κB, IRF, and MAPK inflammatory pathways, we envision this dimer strategy to broaden the use of SMIPs both as adjuvants and in immunotherapy formulations.

## Materials and Methods

Complete details of reagents and methods for cell culture, chemical synthesis, and cell assays are provided in the Supporting Information Materials and Methods section.

## Supporting Information Available

Synthesis of conjugated agonists and dimers, characterization, and additional cell assay data (PDF)

## Abbreviations

NF-κB: nuclear factor kappa-light-chain-enhancer of activated B cells
IL-6: interleukin 6
TNF-α: tumor necrosis factor alpha
SMIPs: small molecule immune potentiators
TLR: toll-like receptor
APC: antigen-presenting cells
