# Supplementary material for: Improving the Adjuvanticity of Small Molecule Immune Potentiators Using Covalently Linked NF-κB Modulators
Source: ACS Med Chem Lett. 2021 Aug 26;12(9):1441–8. doi: 10.1021/acsmedchemlett.1c00267 (PMC8436408; doi:10.1021/acsmedchemlett.1c00267)
Supplement: Supplementary file 1 — ml1c00267_si_001.pdf [file ml1c00267_si_001.pdf]

Supporting Information

Improving the adjuvanticity of small molecule immune potentiators using covalently linked NFκB modulators

Flora W. Kimani, [a] ‡ Saikat Manna, [a] ‡ Brittany Moser, [a] Jingjing Shen, [a] Naorem Nihesh, [a] and Aaron P. Esser-Kahn*[a]

*E-mail: aesserkahn@uchicago.

[a] Pritzker School of Molecular Engineering, University of Chicago, Chicago, IL 60637, United States

Contents

Experimental Procedures S2

General materials and methods S2

Biological Procedures S2

1. RAW264.7 Macrophage (RAW-Blue) NF-κB assay S2
2. Bone Marrow-Derived Dendritic Cell Harvest and Culture S2
3. Western blot analysis of pathway proteins S3
4. Flow Cytometry for Cell Surface Marker Upregulation and Cytokine secretion analysis. S3
5. *In vivo* vaccination of mice S3
6. Plasma cytokine analysis and Antibody quantification S3
7. Tumor studies S3
8. Nitric oxide assay S4
9. Reactive oxygen species measurement S4
10. Proliferation Assay S4
11. Cell viability (MTT) assay S4

Experimental and spectroscopic data of synthesized compounds S5

Supplementary Figures S10

References S12

Experimental Procedures

General materials and methods

Reagents and solvents were purchased from commercial sources and used without further purification. Vanillin, dopamine hydrochloride, 4-hydroxy-3-methoxybenzylamine hydrochloride and ferulic acid were commercially obtained. APC anti-mouse CD40, PE anti-mouse CD11c and purified anti-mouse CD16/32 were purchased from BioLegend. COX-2 (mouse) Polyclonal Antibody was purchased from Cayman Chemical. GAPDH (14C10) Rabbit mAb and iNOS (D6B6S) Rabbit mAb were purchased from Cell Signaling Technology. Spectroscopic characterization was done on Bruker Avance III HD 500 11.7 Tesla NMR (500 MHz) for ^{1}H and ^{13}C NMR. NMR spectra were analyzed using MestreNova software. Coupling on the spectra is expressed in hertz and abbreviations for multiplicities given as s = singlet, d = doublet, t = triplet, dd = doublet of doublets, and m = multiplet where applicable. Mass spectral analysis was performed on Agilent 6224 TOF-MS. Preparative reversed-phase HPLC purification was carried using Phenomenex Luna C18 or C8 Prep (150 X 21.2250 mm, 5 µm particle size) column with a flow rate of 21.2 mL/min on a Gilson 333/334 pump system and GX-271 liquid handler system. UV detection (214 nm, 254 nm, and 260 nm) was used for preparative HPLC. Flow Cytometry data was acquired on a NovoCyte Benchtop Flow Cytometer. Absorbance measurements were acquired on a Multiskan FC plate reader (Thermo Scientific). Data was analyzed using one-way ANOVA in Graph Pad Prism software. All values were reported as mean ± SD. Female C57/BL6 mice and male Balb/c mice were purchased from Jackson Laboratories and allowed to equilibrate for a minimum of 48 h before use. For all experiments, the mice were 6-10 wk old. All animal studies and mice maintenance were approved by the Institutional of Animal Care and Use (IACUC #2012-3048).

Biological Procedures

1. RAW264.7 Macrophage (RAW-Blue) NF-κB assay

RAW-Blue cells, (InvivoGen) were cultured as described by the manufacturer. Cells were grown in complete culture media composed of Dulbecco's Modified Eagle's Medium (DMEM) with 4.5 g/L glucose (Life Technologies), 2 mM L-glutamine, 10,000 U/mL penicillin, 10 mg/mL streptomycin, 25µg/mL amphotericin B, supplemented with 10% fetal bovine serum (FBS, Thermo Fisher Scientific). RAW-Blue cells (passage 5-15) were plated in a 96 well plate at a density of 100,000 cells/well in 180 µL DMEM containing 10% heat-inactivated FBS (HI-FBS) and selective antibiotics. The cells were treated with agonist and agonist dimers and LPS control (50 ng/mL) for 20 h at 37 °C and 5% CO_2. NF-κB activity was measured by a QUANTI-Blue (InvivoGen) assay and the absorbance was measured at 620 nm using a Multiskan FC plate reader (Thermo Scientific).

2. Bone Marrow-Derived Dendritic Cell Harvest and Culture.

Bone marrow-derived dendritic cells (BMDCs) were harvested from female C57Bl/6 mice as previously described.[1] Femur bones were aseptically removed from mice and the bone marrow was extracted into PBS buffer and the cell suspension centrifuged at 300 RCF for 10 min at RT to pellet the cells. ACK Lysing Buffer (3 mL, Lonza) was added to the cell pellet and incubated for 2 min at RT. PBS buffer (13 mL) was then added to the cell suspension, and the cell solution was centrifuged at 300 RCF for 10 min at RT. Next, the cell pellet was resuspended in BMDC complete media (RPMI 1640, 10% heat-inactivated FBS, 20 ng/mL granulocyte-macrophage colony-stimulating factor (GM-CSF), 2 mM L-glutamine (Life Technologies), 10,000 U/mL penicillin, 10 mg/mL streptomycin, 25µg/mL amphotericin B, and 50 µM beta-mercaptoethanol). The cells were then plated at $1x10^{6}$ cells/mL in 100 mm petri dishes in 10 mL complete media and incubated at 37 °C in a CO_2 incubator. On day 3, 10 mL of fresh BMDC media was

added to each petri dish. On day 6, BMDCs were released and plated in untreated 12-well plates at $1x10^6$ cells/mL for cell surface marker activation and cytokine secretion experiments.

3. Western blot analysis of pathway proteins

RAW264.7 Macrophage cells (InvivoGen) cultured in complete media were plated in 6 well plates at 1 $x10^6$ cells/mL and allowed to adhere for 12 h at 37 °C in a CO_2 incubator. The cells were then treated with agonist, agonist dimers, and LPS control for 16 h. The treated cells were washed and scraped into cold phosphate-buffered saline (PBS) and centrifuged at 400 × g at 4°C for 5 min. The cell pellets were resuspended in triple detergent lysis buffer (10 mL) containing one protease inhibitor cocktail (cOmplete™ ULTRA Tablets, Sigma) and centrifuged to yield whole cell lysate. The lysate was quantified using a Pierce™ BCA Protein Assay Kit. 50 µg of total protein was separated using 4-15% SDS–PAGE and blotted onto PVDF membranes (Bio-Rad). The membranes were probed using monoclonal antibodies for COX-2 at a dilution of 1:1000 (Cayman Chemicals, MI) GAPDH (14C10) at a dilution of 1:1000 and Rabbit mAb and at a dilution of 1:500 iNOS (D6B6S) Rabbit mAb Visualization was achieved using IRDye® 800CW (Abcam) at a dilution of 1:10000 and imaged on Azure biosystems imager. Densitometric analysis was done using Image J.

4. Flow Cytometry for Cell Surface Marker Upregulation and Cytokine secretion analysis.

BMDCs were plated in untreated 12-well plates at $1x10^6$ cells/mL and incubated with agonist and agonist dimers in culture media for 8 h at 37 °C with 5% CO2. The cells were released from the plate by pipetting vigorously and centrifuged at 2500 RPM at 4 °C for 10 min. The cell culture media was saved for IL-6 cytokine quantification using ELISA (BioLegend). The cell pellet was resuspended in cold FACS buffer (PBS, 10% FBS, and 0.1% sodium azide) buffer (300 µL) and incubated with CD16/32 FcR blocking antibodies (1.0 µg/1x106 cells) on ice for 15 min. The cell suspension was pelleted, and the supernatant was removed. Next, the cell pellet was resuspended in cold FACS buffer (100 µL) and incubated with PE-CD11c (1.0µg/1x106 cells) and APC CD40 (1.0 µg/1x106 cells), on ice and in the dark for 30 min. The samples were then washed twice with 300 µL FACS buffer. The pelleted cells were resuspended in cold FACS buffer (200 µL) and kept on ice until being loaded onto the flow cytometer for analysis.

5. *In vivo* vaccination of mice

Female C57/BL6 mice were briefly anesthetized with isoflurane and injected intramuscularly in the right hind leg with 50 µL containing ovalbumin (100 µg), adjuvant, adjuvant dimers, vanillin, and dopamine (0.07 µmoles) and a PBS vehicle control group.

6. Plasma cytokine analysis and Antibody quantification

Mouse blood was collected via the submandibular vein in 0.2 mL heparin-coated collection tubes (VWR Scientific) 1 h after vaccination. Serum was isolated by allowing blood to clot for 30 min RT and centrifugation at 2000 x g for 10 min. Supernatant was collected and stored at -80 °C until use. Serum was analyzed using BD Cytometric Bead Array Mouse Inflammation cytokine kit or LEGENDplex™ Mouse Inflammation Panel (Biolegend) according to manufacturer's protocol. For antibody quantification, mouse blood was collected via cardiac puncture 28 days after vaccination in 0.2 mL heparin-coated collection tubes (VWR Scientific). Serum was isolated by allowing blood to clot for 30 min RT and centrifuging at 2000 x g for 10 min. Serum was analyzed using a quantitative anti-ovalbumin total Ig's, IgA, and IgG ELISA kits (Alpha Diagnostic International) according to the manufacture's protocol.

7. Tumor studies

0.2×10^6 CT-26 cells were injected subcutaneously into the flank of 6-week-old Balb/c mice (n = 10 per group) in 100 µL of PBS. The tumor size was monitored on alternating days. Tumor volumes were

measured using the equation V =1/2×L×W×W. When the tumors reached a size of approximately 75 mm^3 (day 11), treatment was started. Various formulations (20 nmols of each agonist or PBS) were injected peritumorally every 4 days (day 15, 19, and 23). Mice were euthanized when the tumors reached 20 mm in any linear dimension. Five mice for each group were used for blood analysis. Blood was collected two days post the first injection for hematological toxicity analysis and two hours post the second injection for systemic cytokine analysis.

8. Nitric oxide assay

RAW264.7 Macrophage (InvivoGen) cultured in complete media were plated in 12-well plates at 1 $x10^6$ cells/mL and treated with agonist and agonist dimers for 16 h at 37 °C in a CO_2 incubator. The cell supernatant was collected and the quantity of nitrite in the culture medium was measured as an indicator of NO production. Amounts of nitrite, a stable metabolite of NO, were measured using Griess reagent (1% sulfanilamide and 0.1% naphthyl ethylenediamine dihydrochloride in 2.5% phosphoric acid). 100 µL of cell culture medium was mixed with 100 µL of Griess reagent. After incubation at room temperature for 10 min, the absorbance at 540 nm was measured in a microplate reader. The quantity of nitrite was determined from a sodium nitrite standard curve.

9. Reactive oxygen species measurement

RAW264.7 Macrophage (InvivoGen) cultured in complete media, were plated in 12-well plates at 1 $x10^6$ cells/mL and treated with agonist and agonist dimers for 16 h at 37 °C in a CO_2 incubator. The cells were released from the plate and centrifuged at 2500 RPM at room temperature for 10 min and the supernatant aspirated. The cells were then washed twice with PBS (200 uL) and the cell pellet resuspended in Hanks' Balanced Salt solution (HBSS) containing CM-H2DCFDA (1uM). Next, the cells were incubated for 30 min at 37 °C with 5 % CO_2. After incubation, the cells were washed twice with cold PBS. Fluorescence was measure using Flow Cytometry on FL-1 (fitc) channel.

10. Proliferation Assay

Proliferation assay was performed as previously reported.[2] Splenocytes were isolated from C57BL/6 mice and plated at 5×10^4 in 96-well plates. The splenocytes were then incubated with 3 µM of agonist and agonist dimers for 48 h at 37 °C in a CO_2 incubator. 3-[4,5-dimethylthiazol-2-yl]-2,5-diphenyltetrazolium bromide (MTT) (20 µl, 5 mg/ml in PBS) was added 4 h before the end of the incubation period. Purple crystals were dissolved in sterile DMSO and incubated for 5 minutes to ensure complete dissolution. The absorbance was measured at 590 nm using a Multiskan FC plate reader (Thermo Scientific). The proliferation rate was determined as follows: Abs(sample)/Abs(PBS)×100%.

11. Cell viability (MTT) assay

RAW264.7 Macrophage cells were incubated with agonist and agonist dimers for 16 h and subjected to cell viability assays. 3-(4,5-dimethylthiazol-2-yl)-2,5-diiphenyltetrazolium bromide (MTT) was dissolved in PBS to final concentration of 5 mg/mL and sterile-filtered. Treated cells were resuspended in fresh RPMI medium with 10 % FBS at concentration and plated in a 96-well plate at a concentration of 1 x 10^5 cell/mL. To these cells, 10 µL of MTT solution was added, then incubated at 37 °C with 5 % CO_2 for 3 h. When purple crystals were visible, 75 µL of supernatant was removed. Purple crystals were dissolved in sterile DMSO and incubated for 5 minutes to ensure complete dissolution. The absorbance was measured at 590 nm using a Multiskan FC plate reader (Thermo Scientific). %viability was calculated as follows: Abs(sample)-Abs(blank)/Abs(Rest cells)-Abs(blank) x 100%.

Experimental and spectroscopic data of synthesized compounds

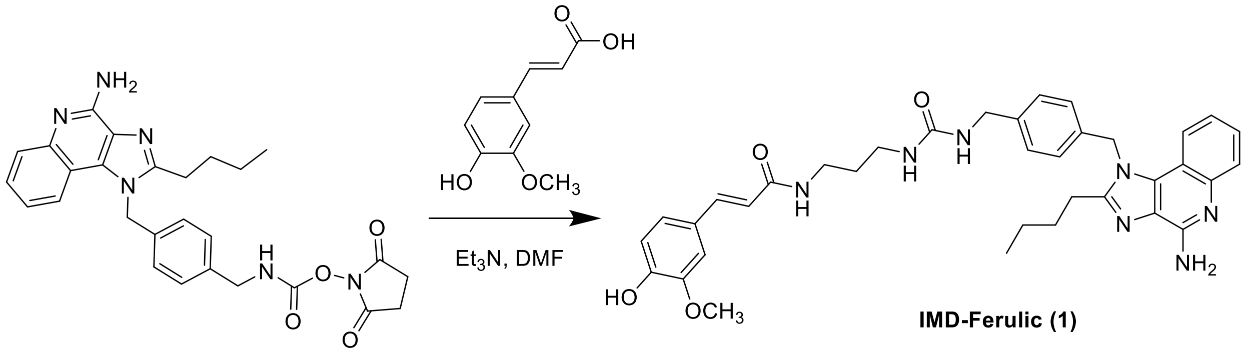

Compound **1** was synthesized as follows. To a solution of **7** (19 mg, 0.078 mmol) and triethylamine (0.01 mL, 0.078 mmol) was added **12** (26 mg, 0.052 mmol) in 1 mL DMF. The mixture was stirred at room temperature under argon for 12 h. The reaction mixture purified using preparative HPLC to give the product as a white solid (15 mg, 40% yield) ^{1}H NMR (400 MHz, DMSO) δ 13.47 (s, 1H), 7.95 (t, *J* = 8.3 Hz, 2H), 7.80 (d, *J* = 8.0 Hz, 1H), 7.62 (t, *J* = 7.4 Hz, 1H), 7.38 (t, *J* = 7.3 Hz, 1H), 7.30 (d, *J* = 15.7 Hz, 1H), 7.20 (t, *J* = 7.6 Hz, 2H), 7.12 (d, *J* = 1.7 Hz, 1H), 7.05 – 6.95 (m, 3H), 6.79 (d, *J* = 8.1 Hz, 1H), 6.42 (d, *J* = 15.7 Hz, 1H), 6.37 (s, 1H), 5.96 (d, *J* = 11.2 Hz, 1H), 5.93 (s, 2H), 4.15 (s, 2H), 3.80 (s, 3H), 3.14 (dd, *J* = 12.6, 6.5 Hz, 2H), 2.98 (dd, *J* = 16.6, 9.0 Hz, 4H), 1.73 (dt, *J* = 15.3, 7.6 Hz, 2H), 1.58 – 1.45 (m, 2H), 1.39 (dq, *J* = 14.7, 7.4 Hz, 2H), 0.87 (t, *J* = 7.4 Hz, 3H) (not observed NH_2). ^{13}C NMR (100 MHz, DMSO) δ 165.8, 158.9, 158.6, 158.5, 157.4, 149.2, 148.7, 148.3, 141.2, 139.3, 135.8, 134.3, 134.1, 129.9, 128.0, 126.9, 125.9, 125.2, 125.1, 122.0, 119.4, 119.0, 116.0, 114.9, 112.9, 111.1, 55.9, 43.9, 36.7, 30.7, 29.7, 29.4, 26.6, 22.9, 22.2, 14.1. HRMS (ESI) m/z calcd for $(C_{36}H_{41}N_7O_4)+H^+$: 636.3298 $[M + H]^+$; found: 636.3294

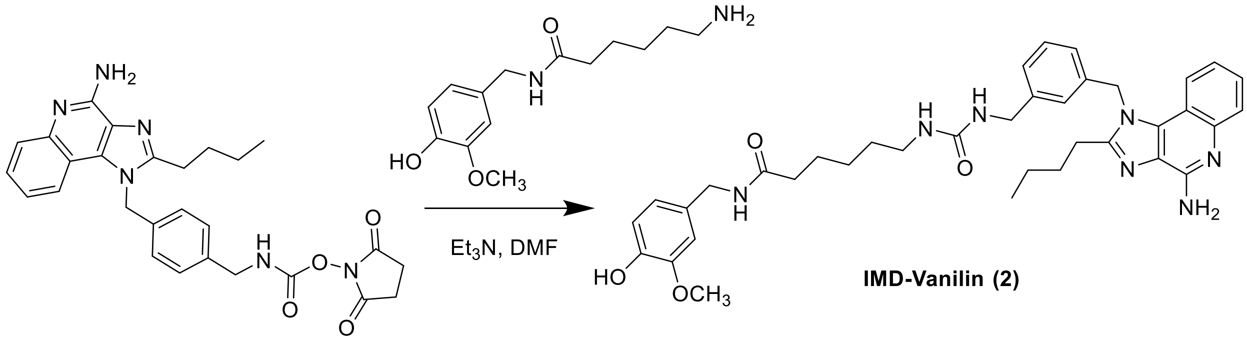

Compound **2** was synthesized as follows. To a solution of **8** (20 mg, 0.066 mmol) and triethylamine (0.01 mL, 0.078 mmol) was added **12** (26 mg, 0.052 mmol) in 1 mL DMF. The mixture was stirred at room temperature for 12 h. The reaction mixture purified using preparative HPLC to give the product as a white solid (18 mg, 54% yield) ^{1}H NMR (400 MHz, DMSO) δ 13.51 (s, 1H), 8.15 (t, *J* = 5.8 Hz, 1H), 7.95 (d, *J* = 8.2 Hz, 1H), 7.79 (d, *J* = 8.0 Hz, 1H), 7.62 (t, *J* = 7.4 Hz, 1H), 7.37 (t, *J* = 7.4 Hz, 1H), 7.18 (t, *J* = 7.3 Hz, 2H), 7.01 (d, *J* = 8.1 Hz, 2H), 6.79 (d, *J* = 1.7 Hz, 1H), 6.69 (d, *J* = 8.0 Hz, 1H), 6.62 (dd, *J* = 8.0, 1.8 Hz, 1H), 6.24 (s, 1H), 5.93 (s, 2H), 5.89 (s, 1H), 4.13 (d, *J* = 5.5 Hz, 4H), 3.72 (s, 4H), 2.95 (dd, *J* = 15.7, 8.0 Hz, 4H), 2.08 (dd, *J* = 9.4, 5.3 Hz, 2H), 1.72 (dt, *J* = 15.3, 7.6 Hz, 2H), 1.49 (dt, *J* = 15.0, 7.5 Hz, 2H), 1.36 (qd, *J* = 14.9, 7.2 Hz, 4H), 1.20 (dt, *J* = 14.7, 7.3 Hz, 2H), 0.87 (t, *J* = 7.4 Hz, 3H) (not observed NH). ^{13}C NMR (100 MHz, DMSO) δ 172.3, 159.0, 158.7, 158.4, 157.4, 149.3, 147.9, 145.8, 141.2, 135.8, 134.4,

134.1, 130.9, 129.8, 128.0, 125.8, 125.2, 125.1, 122.0, 120.1, 118.9, 115.6, 112.9, 112.1, 55.9, 48.6, 42.9, 42.3, 35.8, 30.2, 29.7, 26.7, 26.5, 25.6, 22.2, 14.1. HRMS (ESI) m/z calcd for $(C_{37}H_{45}N_7O_4)+H^+$: 652.3611 $[M + H]^+$; found: 652.3610

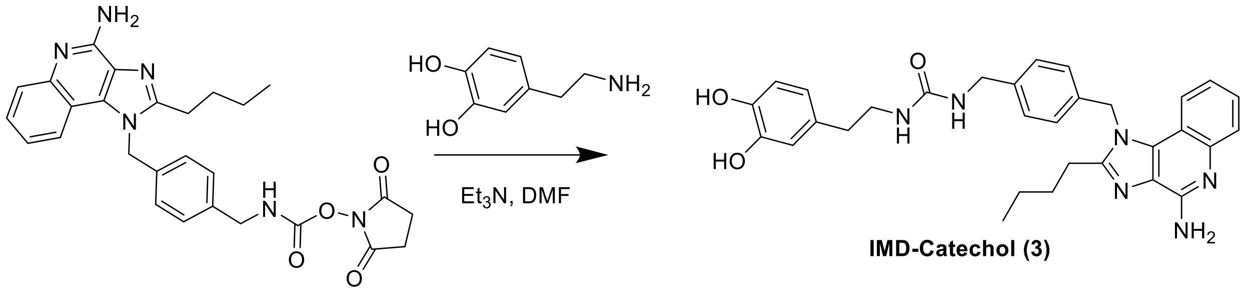

IMD-Catechol (3)

Compound **3** was synthesized as follows. To a solution of dopamine hydrochloride (20 mg, 0.105mmol) and triethylamine (0.027 mL, 0.105 mmol) was added **12** (26 mg, 0.052 mmol) in 1 mL DMF. The mixture was stirred at room temperature for 12 h. The reaction mixture purified using preparative HPLC to give the product as a white solid (9.3 mg, 33% yield) ^1H NMR (400 MHz, DMSO) δ 13.53 (s, 1H), 7.95 (d, *J* = 8.3 Hz, 1H), 7.79 (d, *J* = 8.0 Hz, 1H), 7.62 (t, *J* = 7.8 Hz, 1H), 7.37 (t, *J* = 7.4 Hz, 1H), 7.18 (t, *J* = 6.6 Hz, 2H), 7.01 (d, *J* = 8.1 Hz, 2H), 6.61 (d, *J* = 7.9 Hz, 1H), 6.55 (d, *J* = 1.9 Hz, 1H), 6.40 (dd, *J* = 8.0, 1.9 Hz, 1H), 6.34 (s, 1H), 5.93 (s, 2H), 5.84 (s, 1H), 4.13 (s, 2H), 3.11 (t, *J* = 7.0 Hz, 2H), 3.00 – 2.89 (m, 2H), 2.46 (t, *J* = 7.3 Hz, 2H), 1.73 (dt, *J* = 15.3, 7.6 Hz, 2H), 1.37 (dt, *J* = 14.6, 7.4 Hz, 2H), 0.87 (t, *J* = 7.3 Hz, 3H) (not observed -OH and NH_2). ^{13}C NMR (100 MHz, DMSO) δ 159.1, 158.7, 158.3, 157.4, 149.3, 145.5, 143.9, 141.1, 136.3, 135.8, 134.3, 134.06, 132.28, 130.83, 129.89, 128.06, 125.90, 125.23, 125.13, 122.96, 122.04, 119.67, 118.96, 117.83, 116.4, 115.9, 114.9, 112.9, 48.7, 42.8, 41.7, 36.0, 29.7, 26.7, 22.2, 14.2. HRMS (ESI) m/z calcd for $(C_{31}H_{34}N_6O_4,)$: 554.2642 $[M]^+$; found: 554.2648

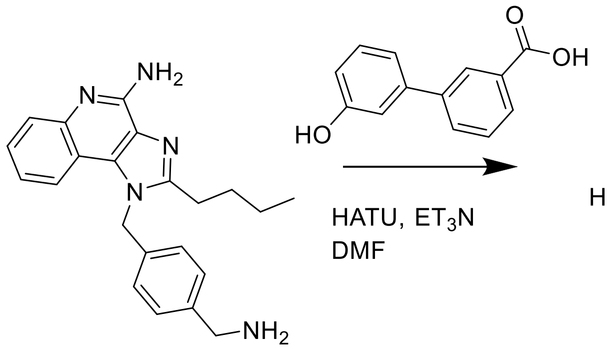

IMD-BiphenylA (4)

Compound **4** was synthesized as follows. To a solution of the imidazoquinoline[3] (30 mg, 0.084 mmol) and **9** (20 mg, 0.093 mmol) in 5 mL DMF stirred at room temperature under argon was added triethylamine (0.015 mL, 1.5 equiv., 0.15 mmol) and 1-[Bis(dimethylamino)methylene]-1H-1,2,3-triazolo[4,5-b]pyridinium 3-oxide hexafluorophosphate (HATU) (41 mg, 1.1 equiv, 0.12 mmol in 0.2 mL DMF). The mixture was stirred at room temperature for 12 h. The reaction mixture was loaded on a silica column and purified by using column chromatography using DCM/MeOH 9:1 to yield an off-white powder. (30 mg, 55% yield) ^1H NMR (400 MHz, DMSO) δ 13.55 (s, 1H), 9.57 (s, 1H), 9.15 (t, J = 5.9 Hz, 1H), 8.09 (s, 1H),

7.97 (d, J = 8.3 Hz, 1H), 7.86 – 7.72 (m, 3H), 7.62 (t, J = 7.7 Hz, 1H), 7.53 (t, J = 7.7 Hz, 1H), 7.37 (t, J = 7.7 Hz, 1H), 7.31 (d, J = 8.4 Hz, 2H), 7.26 (d, J = 7.9 Hz, 1H), 7.12 (d, J = 7.8 Hz, 1H), 7.07 (dd, J = 11.9, 5.1 Hz, 3H), 6.80 (dd, J = 8.0, 2.1 Hz, 1H), 5.95 (s, 2H), 4.47 (d, J = 5.7 Hz, 2H), 3.01 – 2.90 (m, 2H), 1.79 – 1.64 (m, 2H), 1.44 – 1.31 (m, 2H), 0.87 (t, J = 7.3 Hz, 3H). ^{13}C NMR (100 MHz, DMSO) δ 166.5, 158.7, 158.4, 157.8, 149.5, 141.4, 140.8, 139.7, 135.8, 135.2, 134.4, 130.4, 129.8, 129.4, 128.9, 126.8, 126.4, 125.7, 125.1, 122.2, 119.4, 118.3, 115.2, 114.1, 112.9, 48.7, 42.6, 35.5, 32.3, 29.7, 26.6, 22.2, 14.1. HRMS (ESI) m/z calcd for ($C_{35}H_{33}N_5O_2$) +H^+: 556.2713, $[M + H]^+$; found: 556.2718

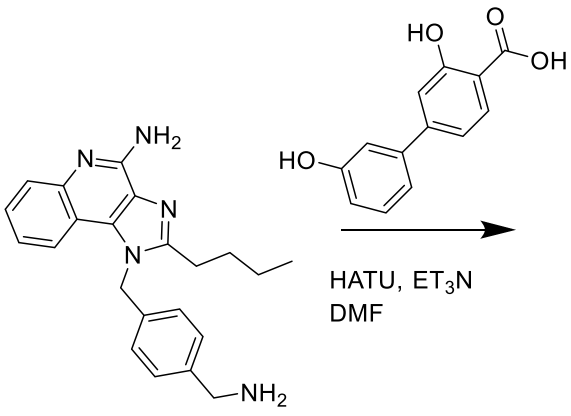

IMD-BiphenylB (5)

Compound **5** was synthesized as follows. To a solution of the imidazoquinoline[3] (30 mg, 0.083 mmol) and **10** (35 mg, 0.15 mmol) in 5 mL DMF stirred at room temperature under argon was added triethylamine (0.015 mL, 1.5 equiv., 0.15 mmol) and HATU (50 mg, 1.1 equiv, 0.13 mmol in 0.2 mL DMF). The mixture was stirred at room temperature for 12 h. The reaction mixture was loaded on a silica column and purified by using column chromatography using DCM/MeOH 9:1 to yield an off-white powder. The product was further purified using preparative HPLC (20 mg, 55% yield) ^{1}H NMR (400 MHz, DMSO) δ 13.89 (s, 1H), 12.55 (s, 1H), 9.35 (t, *J* = 6.0 Hz, 1H), 9.05 (s, 2H), 8.01 – 7.87 (m, 2H), 7.84 – 7.73 (m, 1H), 7.67 – 7.57 (m, 1H), 7.40 – 7.20 (m, 4H), 7.16 – 6.96 (m, 6H), 6.82 – 6.77 (m, 1H), 5.95 (s, 2H), 4.49 (d, *J* = 5.8 Hz, 2H), 3.00 – 2.93 (m, 2H), 1.78 – 1.65 (m, 2H), 1.38 (dq, *J* = 14.7, 7.4 Hz, 2H), 0.90 – 0.82 (m, 3H). ^{13}C NMR (100 MHz, DMSO) δ 169.4, 161.0, 158.4, 157.4, 149.4, 145.9, 139.2, 135.8, 135.0, 134.6, 134.8, 129.9, 129.8, 128.6, 128.5, 128.6, 126.3, 126.1, 125.2, 125.1, 122.0, 118.9, 118.3, 116.8, 116.2, 115.4, 114.4, 113.4, 112.9, 48.7, 42.4, 29.7, 26.7, 22.2, 14.1 HRMS (ESI) m/z calcd for ($C_{35}H_{33}N_5O_3$)+ H^+: 572.2662 $[M + H]^+$; found: 572.2659

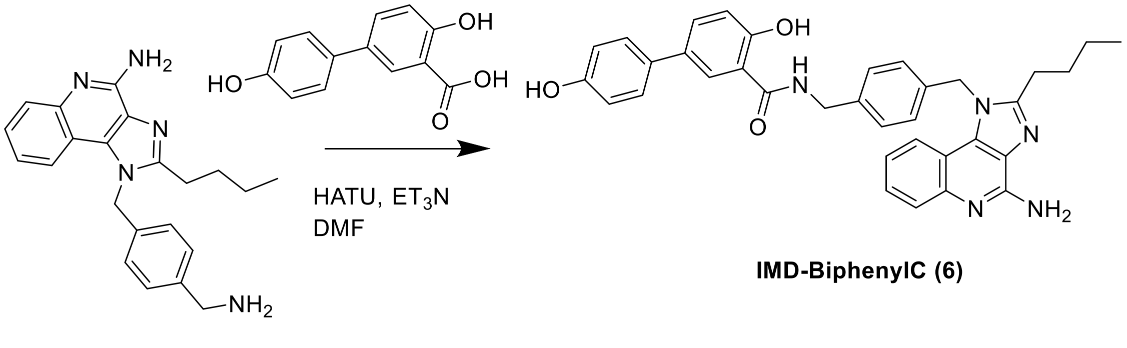

IMD-BiphenylC (6)

Compound **6** was synthesized as follows. To a solution of the imidazoquinoline (ref) (30 mg, 0.084 mmol) and **11** (20 mg, 0.093 mmol) in 5 mL DMF stirred at room temperature under argon was added triethylamine (0.015 mL, 1.5 equiv., 0.15 mmol) and HATU (41 mg, 1.1 equiv, 0.12 mmol in 0.2 mL DMF). The mixture was stirred at room temperature for 12 h. The reaction mixture was loaded on a silica

column and purified by using column chromatography using DCM/MeOH 9:1 to yield an off-white powder. The product was further purified using preparative HPLC (33.3 mg, 75 % yield) ^{1}H NMR (400 MHz, DMSO) δ 13.82 (s, 1H), 12.52 (s, 1H), 9.25 (t, *J* = 6.0 Hz, 1H), 8.97 (s, 2H), 7.90 (d, *J* = 8.3 Hz, 1H), 7.81 (d, *J* = 8.4 Hz, 1H), 7.74 – 7.68 (m, 1H), 7.58 – 7.52 (m, 1H), 7.49 – 7.43 (m, 2H), 7.29 (dd, *J* = 11.4, 4.1 Hz, 1H), 7.23 (d, *J* = 8.2 Hz, 2H), 7.05 (dd, *J* = 8.4, 1.8 Hz, 1H), 7.02 – 6.95 (m, 3H), 6.82 – 6.72 (m, 2H), 5.84 (d, *J* = 27.7 Hz, 2H), 4.40 (d, *J* = 5.7 Hz, 2H), 2.95 – 2.85 (m, 2H), 1.65 (dt, *J* = 15.3, 7.5 Hz, 2H), 1.39 – 1.23 (m, 2H), 0.79 (t, *J* = 7.3 Hz, 3H).^{13}C NMR (100 MHz, DMSO) δ 169.1, 160.7, 158.3, 157.4, 149.4, 146.0, 140.7, 139.1, 135.8, 134.6, 134.8, 130.5, 129.9, 128.9, 128.4, 126.1, 125.2, 125.1, 122.0, 118.9, 118.3, 117.9, 117.6, 115.8, 115.8, 114.6, 113.9, 112.9, 48.7, 42.4, 29.7, 26.7, 22.2, 14.1 HRMS (ESI) m/z calcd for $(C_{35}H_{33}N_5O_3)+H^+$: 572.2662 $[M + H]^+$ found: 572.2659

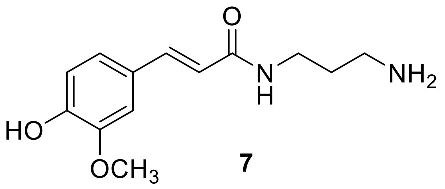

7

Compound **7** was synthesized as follows. To a solution of trans-ferulic acid (200 mg, 1.03 mmol, 1.0 equiv.) and 3-azidopropylamine (0.15 mL, 1.5 mmol, 1.5 equiv.) in 5 mL DMF, was added triethylamine (0.14 mL, 1.02 mmol, 1.0 equiv.) and HATU (390 mg, 1.02 mmol, 1.0 equiv.) and the solution stirred for 12 h at RT under argon. The reaction mixture was extracted into ethyl acetate (10 mL X 3) and the solvent evaporated in vacuo. The crude product obtained from the organic layer was dissolved in 5 mL MeOH/H2O 4:1 and to this solution Tris(2-carboxyethyl) phosphine hydrochloride (295 mg, 1.03 mmol) was added and the mixture stirred for 12 h at RT. The reaction mixture was loaded on a silica column and purified by using column chromatography using DCM/MeOH 9:1 to yield an amorphous solid (80 mg, 67 % yield). ^{1}H NMR (400 MHz, DMSO) δ 8.20 (t, *J* = 5.8 Hz, 1H), 7.77 (d, *J* = 39.7 Hz, 2H), 7.33 (d, *J* = 15.7 Hz, 1H), 7.13 (d, *J* = 1.7 Hz, 1H), 6.99 (dd, *J* = 8.2, 1.7 Hz, 1H), 6.79 (d, *J* = 8.1 Hz, 1H), 6.45 (d, *J* = 15.7 Hz, 1H), 3.80 (s, 3H), 3.24 (q, *J* = 6.5 Hz, 2H), 2.81 (dt, *J* = 12.4, 6.1 Hz, 2H), 1.78 – 1.68 (m, 2H) (not observed OH). ^{13}C NMR (100 MHz, DMSO) δ 166.4, 148.8, 148.3, 139.7, 126.7, 122.10, 119.0, 116.1, 111.2, 55.9, 37.3, 36.2, 28.0. HRMS (ESI) m/z calcd for $(C_{13}H_{18}N_2O_3)+Na^+$: 273.1215 $[M + Na]^+$; found: 273.1209

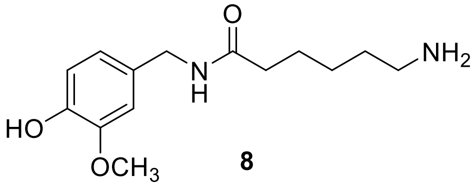

8

Compound **8** was synthesized as follows. To a solution of 4-hydroxy-3-methoxybenzylamine hydrochloride (75 mg, 0.40 mmol, 1.0 equiv.) and 6-Azidohexanoic Acid NHS ester (100 mg, 0.40 mmol, 1.0 equiv.) in 1 mL DMF was added triethylamine (0.06 mL, 0.43 mmol, 1.2 equiv.) and the solution stirred for 12 h at RT under argon. The reaction mixture was dissolved in 5 mL MeOH/H2O 4:1 and to this solution Tris(2-carboxyethyl) phosphine hydrochloride (120 mg, 0.40 mmol) was added and the mixture stirred for 12 h at RT. The reaction mixture was loaded on a silica column and purified by using column chromatography using DCM/MeOH 9:1 to yield an amorphous solid (58 mg, 50 % yield). ^{1}H NMR (400 MHz, DMSO) δ 8.84 (s, 1H), 8.19 (t, *J* = 5.7 Hz, 1H), 7.68 (s, 2H), 6.81 (d, *J* = 1.7 Hz, 1H), 6.70 (d, *J* = 8.0 Hz, 1H), 6.63 (dd, *J* = 8.0, 1.8 Hz, 1H), 4.15 (d, *J* = 5.8 Hz, 2H), 3.74 (s, 3H), 2.84 – 2.70 (m, 2H), 2.17 – 2.06 (m, 2H), 1.59 – 1.44 (m, 4H), 1.36 – 1.23 (m, 2H).^{13}C NMR (100 MHz, DMSO) δ 172.2, 147.9, 145.8, 130.9, 120.2, 115.6, 112.2, 56.0, 42.3, 35.6, 27.3, 25.9, 25.3. HRMS (ESI) m/z calcd for $(C_{14}H_{22}N_2O_3)+Na^+$: 289.1528 $[M + Na]^+$; found: 289.1521

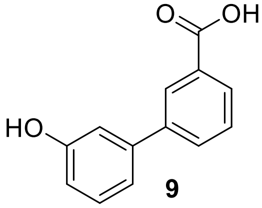

9

Compound **9** was synthesized as follows. A mixture of 3-hydroxyphenylboronic acid (220 mg, 1.59 mmol) 3-iodobenzoic acid (200 mg, 0.81 mmol) potassium carbonate (400 mg, 2.89 mmol) and Pd/C (10%) in 20 mL H2O was refluxed at 80 °C for 4 h. Solution was acidified with 1M HCl and extracted with ethylacetate and washed with brine. Solvent evaporated in vacuo. Compound was purified by column chromatography to yield product as white powder (120 mg, 69% yield) ^{1}H NMR (400 MHz, DMSO) δ 8.13 (t, J = 1.6 Hz, 1H), 7.96 – 7.91 (m, 1H), 7.88 – 7.82 (m, 1H), 7.58 (dd, J = 9.6, 5.8 Hz, 1H), 7.28 (t, J = 7.9 Hz, 1H), 7.13 – 7.05 (m, 2H), 6.81 (dd, J = 7.8, 2.0 Hz, 1H) (not observed OH, COOH). ^{13}C NMR (100 MHz, DMSO) δ 167.7, 158.4, 141.1, 141.1, 131.9, 131.4, 130.6, 129.8, 128.6, 127.6, 117.9, 115.4, 113.9. HRMS (ESI) m/z calcd for ($C_{13}H_{10}O_3$): 214.0630 $[M]^+$; found: 214.0673

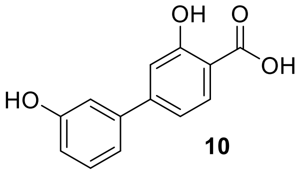

10

Compound **10** was synthesized as follows. A mixture of 3-hydroxyphenylboronic acid (220 mg, 1.59 mmol) 2-hydroxy-4-iodobenzoic acid (210 mg, 0.79 mmol) potassium carbonate (400 mg, 2.89 mmol) and Pd/C (10%) in 20 mL H2O was refluxed at 80 °C for 4 h. Solution was acidified with 1M HCl and extracted with ethylacetate and washed with brine. Solvent evaporated in vacuo. Compound was purified by column chromatography to yield product as white powder (126 mg, 68%) ^{1}H NMR (400 MHz, DMSO) δ 13.97 (s, 1H), 11.33 (s, 1H), 9.56 (d, *J* = 40.7 Hz, 1H), 7.83 (dd, *J* = 20.0, 8.2 Hz, 1H), 7.28 (t, *J* = 7.9 Hz, 1H), 7.15 (ddd, *J* = 14.2, 10.4, 5.0 Hz, 3H), 7.05 (dd, *J* = 5.2, 3.2 Hz, 1H), 6.86 – 6.80 (m, 1H).^{13}C NMR (100 MHz, DMSO) δ 172.2, 161.8, 158.3, 158.1, 147.8, 140.5, 131.3, 130.5, 118.1, 116.0, 115.0, 114.1, 112.3. HRMS (ESI) m/z calcd for ($C_{13}H_{10}O_4$): 230.0579 $[M]^+$; found: 230.0551

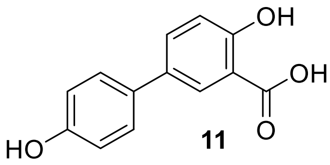

11

A mixture of 4-hydroxyphenylboronic acid (220 mg, 1.59 mmol) 3-iodobenzoic acid (200 mg, 0.79 mmol) potassium carbonate (400 mg, 2.89 mmol) and Pd/C (10%) in 20 mL H2O was refluxed at 80 °C for 4 h. Solution was acidified with 1M HCl and extracted with ethylacetate and washed with brine. Solvent evaporated in vacuo. Compound was purified by column chromatography to yield product as white powder (75 mg, 41% yield) ^{1}H NMR (400 MHz, DMSO) δ 11.19 (s, 1H), 9.51 (s, 1H), 7.93 (t, *J* = 5.3 Hz, 1H), 7.74 (dd, *J* = 8.6, 2.5 Hz, 1H), 7.43 (ddd, *J* = 7.7, 4.8, 2.4 Hz, 3H), 7.01 (d, *J* = 8.6 Hz, 1H), 6.86 – 6.81 (m, 2H). ^{13}C NMR (100 MHz, DMSO) δ 172.3, 160.2, 157.2, 133.8, 132.0, 130.3, 127.7, 127.6, 127.5, 118.1, 116.2, 116.2, 113.6. HRMS (ESI) m/z calcd for ($C_{13}H_{10}O_4$)+H^+: 231.0657 $[M + H]^+$ found: 231.0673

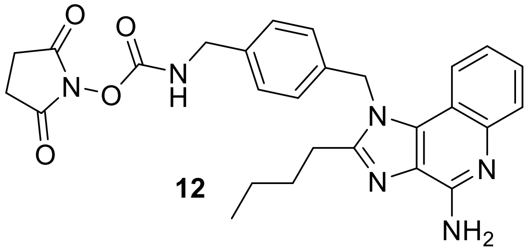

12

Compound **12** was synthesized as follows. To a solution of imidazoquinoline[3] (60 mg, 0.167mmol) and triethylamine (0.023, 0.167 mmol) was added N,N′-Disuccinimidyl carbonate (60 mg, 0.235 mmol) was added. The reaction mixture was stirred at room temperature for 6 h. The reaction mixture was loaded on a silica column and purified by using column chromatography using DCM/MeOH 9:1 to yield a low melting clear solid (40 mg, 60 % yield) ^{1}H NMR (500 MHz, DMSO) δ 8.85 (t, J = 6.0 Hz, 1H), 7.96 (d, J = 8.3 Hz, 1H), 7.79 (d, J = 8.3 Hz, 1H), 7.69 – 7.55 (m, 1H), 7.36 (dd, J = 21.9, 14.0 Hz, 1H), 7.22 (dd, J = 16.2, 8.0 Hz, 2H), 7.05 (dd, J = 22.6, 7.6 Hz, 2H),

5.95 (s, 2H), 4.23 (d, J = 3.6 Hz, 2H), 2.96 (t, J = 7.7 Hz, 2H), 2.75 (s, 4H), 1.73 (m, J = 15.2, 7.7 Hz, 2H), 1.38 (m, J = 14.6, 7.4 Hz, 2H), 0.87 (dd, J = 7.8, 6.9 Hz, 3H) (not observed NH_2). ^{13}C NMR (125 MHz, DMSO) δ 171.3, 157.5, 152.6, 148.7, 146.0, 138.3, 135.9, 134.9, 129.9, 128.2, 126.2, 125.2, 125.1, 122.5, 118.9, 112.8, 48.7, 44.4, 29.6, 26.6, 25.7, 22.2, 14.1.HRMS (ESI) m/z calcd for $(C_{27}H_{28}N_6O_4)+H^+$: 501.2250 $[M + H]^+$ found: 501.2261

Supplementary Figures

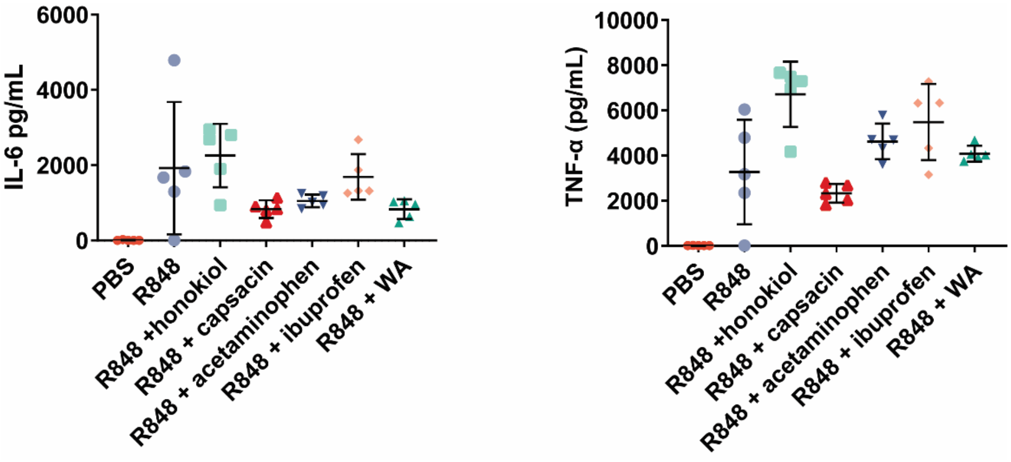

Figure S1. In vivo assays with R848 and NF-κB inhibitor. Serum levels of cytokines assayed 1h after injection. IL-6 and TNF- α levels are not significantly reduced when compared with R848.

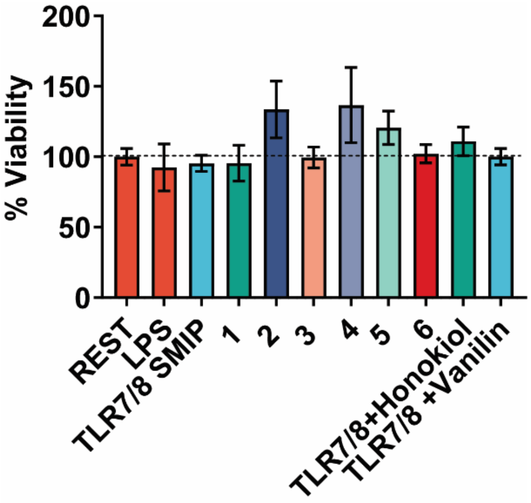

Figure S2. MTT assay showing the viability of agonist and agonist dimer treated cells. At the assayed concentrations the cells have comparable viability to resting cells.

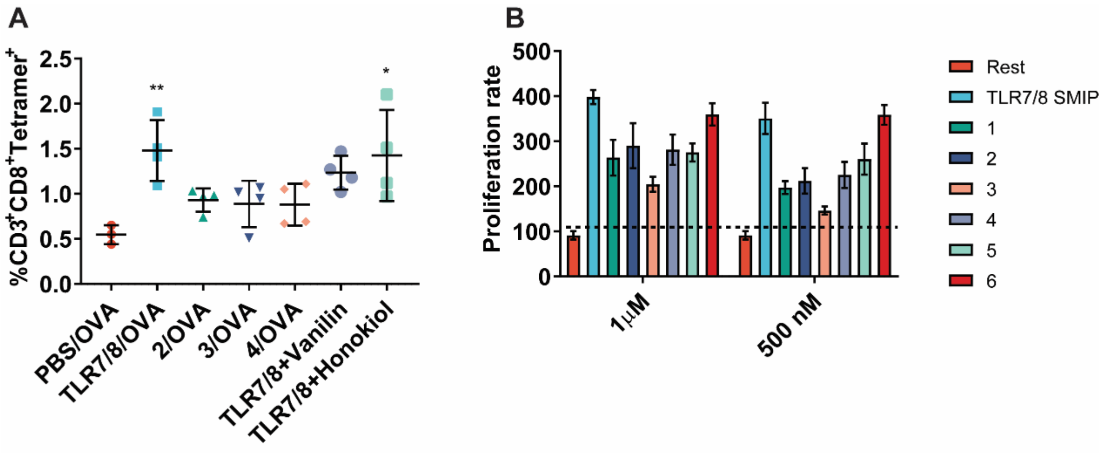

Figure S3. A) SINFEKL MHC-specific tetramer on day 28 post-vaccination isolated spleens. B) Proliferation assay on naïve spleenocytes

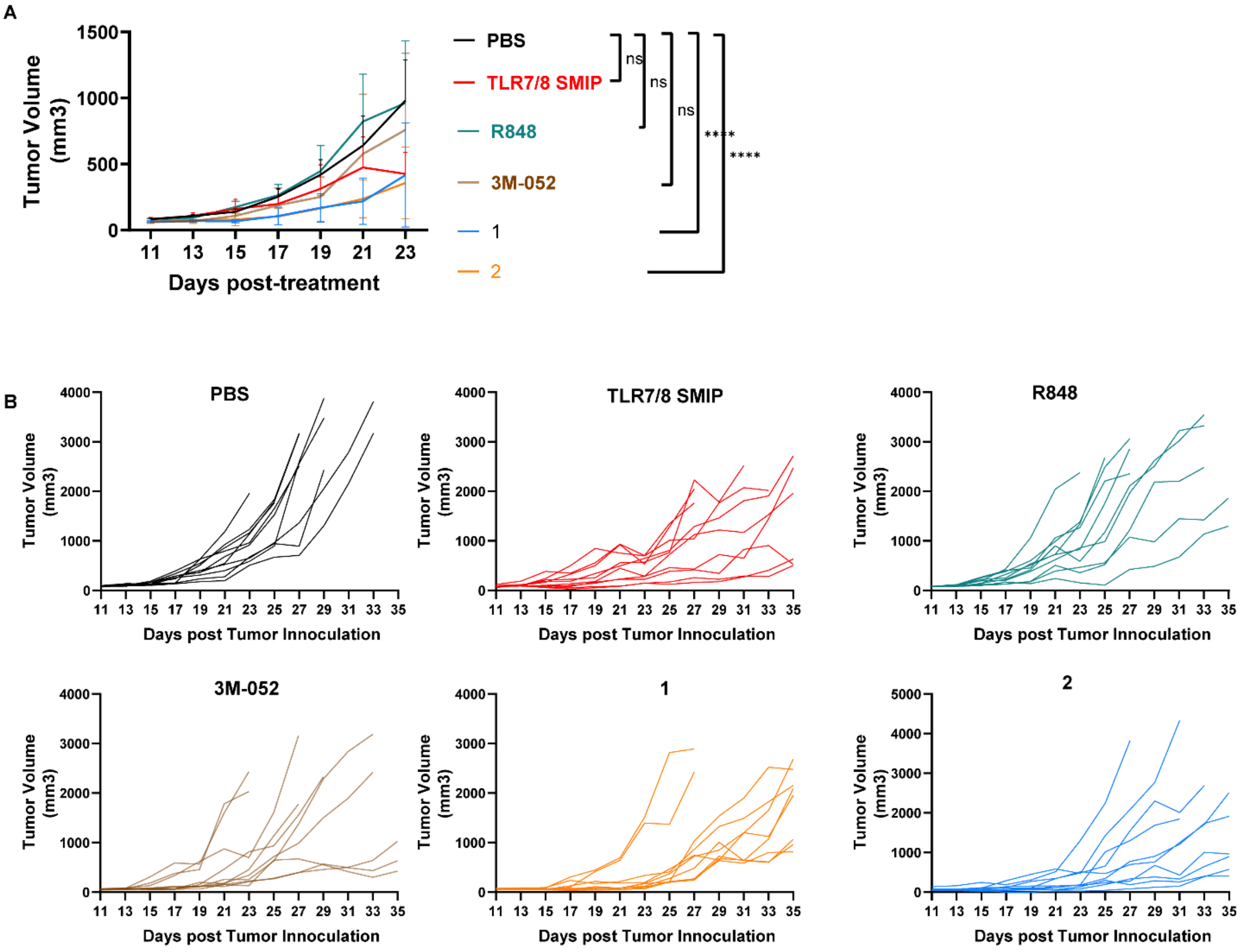

Figure S4. *In vivo* tumor model experiment using SMIP-modulator dimers with peritumoral injection into subcutaneous CT-26 tumor model. Agonists were injected when tumors were about 75 cc in size followed by three additional injections every four days.

References

(1) Tom, J. K.; Dotsey, E. Y.; Wong, H. Y.; Stutts, L.; Moore, T.; Davies, D. H.; Felgner, P. L.; Esser-Kahn, A. P. Modulation of Innate Immune Responses via Covalently Linked TLR Agonists. *ACS Cent. Sci*. **2015**, *1* (8), 439–448

(2) Ma, F.; Zhang, J.; Zhang, J.; Zhang, C. The TLR7 Agonists Imiquimod and Gardiquimod Improve DC-Based Immunotherapy for Melanoma in Mice. *Cell. Mol. Immunol.* **2010**, *7*, 381–388

(3) Shukla, N. M.; Malladi, S. S.; Mutz, C. A.; Balakrishna, R.; David, S. A. Structure-Activity Relationships in Human Toll-like Receptor 7-Active Imidazoquinoline Analogues. *J. Med. Chem.* **2010**, *53* (11), 4450–4465
